# Redox Balance in *Lactobacillus reuteri* DSM20016: Roles of Iron-Dependent Alcohol Dehydrogenases in Glucose/ Glycerol Metabolism

**DOI:** 10.1371/journal.pone.0168107

**Published:** 2016-12-28

**Authors:** Lu Chen, Paul David Bromberger, Gavin Nieuwenhuiys, Rajni Hatti-Kaul

**Affiliations:** 1 Biotechnology, Center for Chemistry and Chemical Engineering, Lund University, Lund, Sweden; 2 Campus Tulln-Biotechnological processes, University of Applied Sciences Wiener Neustadt, Konrad-Lorenz-Strasse 10, Tulln, Austria; National Renewable Energy Laboratory, UNITED STATES

## Abstract

*Lactobacillus reuteri*, a heterofermentative bacterium, metabolizes glycerol via a Pdu (propanediol-utilization) pathway involving dehydration to 3-hydroxypropionaldehyde (3-HPA) followed by reduction to 1,3-propandiol (1,3-PDO) with concomitant generation of an oxidized cofactor, NAD^+^ that is utilized to maintain cofactor balance required for glucose metabolism and even for oxidation of 3-HPA by a Pdu oxidative branch to 3-hydroxypropionic acid (3-HP). The Pdu pathway is operative inside Pdu microcompartment that encapsulates different enzymes and cofactors involved in metabolizing glycerol or 1,2-propanediol, and protects the cells from the toxic effect of the aldehyde intermediate. Since *L*. *reuteri* excretes high amounts of 3-HPA outside the microcompartment, the organism is likely to have alternative alcohol dehydrogenase(s) in the cytoplasm for transformation of the aldehyde. In this study, diversity of alcohol dehydrogenases in *Lactobacillus* species was investigated with a focus on *L*. *reuteri*. Nine ADH enzymes were found in *L*. *reuteri* DSM20016, out of which 3 (PduQ, ADH6 and ADH7) belong to the group of iron-dependent enzymes that are known to transform aldehydes/ketones to alcohols. *L*. *reuteri* mutants were generated in which the three ADHs were deleted individually. The lagging growth phenotype of these deletion mutants revealed that limited NAD^+^/NADH recycling could be restricting their growth in the absence of ADHs. Notably, it was demonstrated that PduQ is more active in generating NAD^+^ during glycerol metabolism within the microcompartment by resting cells, while ADH7 functions to balance NAD^+^/NADH by converting 3-HPA to 1,3-PDO outside the microcompartment in the growing cells. Moreover, evaluation of ADH6 deletion mutant showed strong decrease in ethanol level, supporting the role of this bifuctional alcohol/aldehyde dehydrogenase in ethanol production. To the best of our knowledge, this is the first report revealing both internal and external recycling for cofactor homeostasis during 3-HPA conversion in *L*. *reuteri*.

## Introduction

*Lactobacillus* species constitute an important group of lactic acid bacteria (LAB) that are normally used as probiotics, for production of fermented foods, and also of biobased chemicals like lactic acid and 1,3-propanediol (1,3-PDO) [1–3]. The production of 1,3-PDO is achieved due to the ability of the bacteria to use glycerol as an indirect electron acceptor that helps to maintain regeneration of cofactor needed for maintaining glucose metabolism, cell growth and energy production [4–8].

*Lactobacillus reuteri* is an obligate heterofermentative bacteria that grows on several carbon sources and is well recognised for its probiotic effect [9]. Metabolic flux analysis has shown that *L*. *reuteri* uses both phosphoketolase pathway (PKP) and Embden-Meyerhof pathway (EMP) for glucose metabolism; the primary flux is through the PKP while the EMP is used as a mere shunt [10]. The organism does not grow on glycerol, but addition of glycerol, 1,2-propanediol or 1,2-ethanediol to the cultivation medium induces the expression of genes in the propanediol-utilization (Pdu) operon encoding shell proteins and enzymes needed for metabolism of glycerol (or the other diols) and use as electron acceptor[11].

The glycerol metabolism is initiated by vitamin B12-dependent glycerol dehydratase (PduCDF) catalysed dehydration to 3-hydroxypropionaldehyde (3-HPA), followed by a reductive and an oxidative route [12]. Reduction of 3-HPA to 1,3-PDO is catalysed by a NAD(P)^+^-dependent alcohol dehydrogenase (PduQ), whereas oxidation to 3-hydroxypropionic acid (3-HP) is catalyzed by a set of 3 enzymes, propionaldehyde dehydrogenase (PduP), phosphotransacylase (PduL) and propionate kinase (PduW) [12–14]. The Pdu structural proteins form microcompartments (MCP) called metabolosomes that encapsulate the components of the metabolic pathways, and are expected to protect the cells against the toxic effect of the intermediate aldehyde, while allowing enzyme substrates (e.g. glycerol), cofactors (e.g. NAD^+^, NADH), and products (e.g. 1,3-PDO, 3-HP) to pass [15].

1,3-PDO is the main product of glycerol metabolism by the growing cells, providing the cofactor needed for glucose metabolism. On the other hand, the resting cells convert glycerol to an equimolar mixture of 1,3-PDO and 3-HP with maintained cofactor recycling, but significant accumulation of 3-HPA occurs at high feeding rate of glycerol [12,16]. 3-HPA forms an equilibrium mixture with 3-HPA hydrate and dimer called as reuterin to which is attributed the probiotic role of *L*. *reuteri* [16]. Our laboratory studies have further shown that unlike 3-HPA, propionaldehyde produced from 1,2-propanediol (1,2-PDO) by *L*. *reuteri*, is not excreted outside of MCP (unpublished data). These observations suggest that *L*. *reuteri* is likely to have other routes for conversion of 3-HPA outside the MCP. Hence, understanding the role of other alcohol dehydrogenases in 3-HPA reduction becomes important.

Alcohol dehydrogenases (ADHs) comprise an extremely diverse group of enzymes catalysing the interconversion of alcohols and aldehydes or ketones [17]. Several categories of ADHs can be analyzed based on their cofactor specificity, these being: (i) NAD^+^ or NADP^+^, (ii) the pyrrolo-quinoline quinine, haem or cofactor F_420_, and (iii) FAD [18]. The NAD(P)-dependent ADHs are further sub-divided into zinc-dependent (group I), short-chain (group II), and iron-dependent (group III) ADHs [19]. Most of the known ADHs from *Lactobacillus sp*. are NAD(P)-dependent, which indicates that they share a common catalytic mechanism. Among the group III NAD(P)-dependent bacterial ADHs that have been described and characterised so far are lactaldehyde dehydrogenases, butanol dehydrogenases, 1,3-propanediol (1,3-PD) dehydrogenases while there are several remaining to be characterized [19,20]. In general, the group III enzymes have been found to preferentially catalyse the reduction of aldehydes.

The current study was initiated to explore the diversity of ADH enzymes in *Lactobacillus* species and *L*. *reuteri* DSM20016 in particular, with a subsequent focus on the iron-dependent group III ADHs and their role in the redox balance in glycerol/glucose metabolism.

## Materials and Methods

### Bacterial strains, plasmids and primers

The bacterial strains and plasmids used and the primers constructed in this study are listed in S1 Table. *Escherichia coli* strains DH5α was used as an intermediate cloning host and was grown at 37°C in Luria Bertani (LB) broth. For preparation of inoculum of *L*. *reuteri*, wild type as well as mutants, 20 mL of 55 g/L MRS broth (Difco) supplemented with 20 mM glycerol was added to the 30 mL serum bottles, boiled, and bubbled with nitrogen gas, after which the bottles were closed with rubber stoppers, and autoclaved at 121°C for 10 min. After inoculation of the medium, the cells were grown anaerobically without shaking for 8 h at 37°C. The stock culture was stored at -80°C in MRS broth containing 20% v/v glycerol. For selective cultivation of the mutants, appropriate antibiotics were supplemented to the media. For recombinant *E*. *coli*, 10 μg/mL chloramphenicol and 250 μg/mL erythromycin were used, while for recombinant *L*. *reuteri* 10 μg/mL chloramphenicol and 30 μg/mL erythromycin were used.

### Chemicals and reagents

All restriction enzymes, X-Gal and isopropyl-β-D-1-thiogalactopyranoside (IPTG) were purchased from Fermentas. T4 DNA ligase and Taq DNA polymerase were from New England Biolabs, while BugBuster protein extraction reagent was from Novagen. β-Nicotinamide adenine dinucleotide, reduced disodium salt hydrate (NADH), β-Nicotinamide adenine dinucleotide (NAD^+^), and all antibiotics were purchased from Sigma-Aldrich. Primers and other kits for DNA extraction and purification were from Thermo Fisher Scientific.

### Bioinformatics analysis

Geneious 9.1.2 was utilized for detection of enzymes in the Protein DataBank having sequence resemblance to ADHs. Multiple sequence alignments for identification of conserved amino acids were analyzed by ClustalW2 tools (EMBL-EBI) and visualized in Geneious. The derived *Lactobacillus* ADH sequences for phylogenetic analysis were extracted from protein database UniProt. Homology modeling of the *L*. *reuteri* ADHs was performed with the aid of Program DeepView (Swiss PDB-Viewer) [21]. Homology model quality assessment was performed by utilizing the SWISS-MODEL workspace. Chimera developed by the UCSF Resource (http://www.cgl.ucsf.edu/chimera) was used as a platform for the study of dockings with NADH using the tool AutoDock Vina [22,23]. Figures were generated using either Chimera or PyMol (http://www.pymol.org) and raytraced images are produced with POV-Ray. Global alignment(Cost Matrix: Blosum 62, Gap open penalty:12, Gap extension penalty:3) was adapted for building distance matrix by using Neighbor-Joining (NJ) as the tree bulid method and Jukes-Cantor as genetic distance model (bootstrap number: 100). No outgroup was chosen for rooting the phylogenetic tree.

### Construction of plasmids and mutant strains

The ADH genes were amplified from the genomic DNA of *L*. *reuteri* DSM20016. The approach to obtain gene deletion variants was based on double-crossover integration method reported earlier by Jolamda et al. [24]. Generally, about 1-kb fragment of the sequences upstream and downstream of the target locus were amplified and cloned into the SwaI and SrfI blunt-end restriction sites of pNZ5319, respectively. Colony PCR was performed to identify the colonies harboring the anticipated insert in the desired orientation.

Using this approach, the *PduQ* mutagenesis vector pLCH007 was constructed by successive cloning of both 5’- and 3’- flanking regions of *PduQ* gene (lreu_1734). Clones that harbored the desired inserts were identified by PCR using primer sets 13–16 (S1 Table). Likewise, vectors pLCH008 and pLCH009 were successfully constructed to harbor the 5’- and 3’- flanking regions of *ADH6* and *ADH7* gene inserts, respectively. Approprate primer sets 17–24 were designed to check the correct insertion.

The gene-specific mutagenesis vectors (1–4 μL) were transformed into *L*. *reuteri* DSM20016 in order to engineer *lox66*-P_32-_*cat-lox71* gene replacement by electroporation as previously described [25], with slight modification. An overnight culture of the transformed *L*. *reuteri* strains was used to inoculate 15 mL MRS broth containing 2% glycine and 0.5 M sucrose, and incubated at 37°C. The absorbance of the culture at 600 nm reached 0.2 after 3 h, after which the cells were separated by centrifugation and then washed twice with 5 mL cold distilled water followed by a 10 min incubation in 50 mM EDTA solution (pH 8.0). After washing with cold distilled water and 0.3 M sucrose, the cells were re-suspended in 100 μL of 0.3 M sucrose solution as electroporation buffer. Then the suspension was transferred into a disposable cuvette (Bio-Rad Laboratories) and subjected to an electric pulse using a MicroPulser (Bio-Rad) under the following conditions: Capacitance = 25 μF; Resistance = 400 ohm; Voltage = 2.0 kV.

Candidate double-crossover clones (Cm^r^), which indicated correct integration of the *lox66*-P_32-_*cat-lox71* into the genome was confirmed by PCR amplification using primer pairs 13:16, 17:20 and 21:24 for *PduQ*, *ADH6* and *ADH7*, respectively.

In order to cut off the P_32_*-cat* cassette from the chromosome, 1–4 μL of *cre* expression vector pNZ5348 was transformed into the mutants. Em^r^ (erythromycin resistance) colonies were tested by PCR for desired *Cre*-mediated recombination, using primer pairs of 25:26, 27:28 and 29:30 for *PduQ*, *ADH6* and *ADH7*, respectively. The pNZ5348 vector was cured from relevant colonies of *L*. *reuteri* mutants by growth without erythromycin selection pressure for about 10 generations.

All the above inserts were verified by DNA sequencing. Other molecular cloning and DNA manipulation procedures, not mentioned here, were essentially done as described by Sambrook et al. [26].

### Recombinant expression and production of *L*. *reuteri* PduQ and ADH7

The gene sequences coding for PduQ and ADH7 genes were amplified by using appropriate primer pairs (S1 Table). Initial denaturing at 95°C for 3 min was followed by 30 cycles of denaturing at 95°C for 30 s, annealing at 54°C for 30 s and elongation at 72°C for 1 min. A final elongation was performed at 72°C for 15 min. The PCR product was separated and purified by agarose gel electrophoresis and inserted directly into the expression vector pET21a after digesting both vector and PCR product with the restriction enzymes BamHI and XhoI. The resulting plasmids pET21a-*pduQ*-His_6_/ pET21a-*ADH7*-His_6_ were introduced in *E*. *coli* BL21(DE3) and the recombinant strains were designated as *E*. *coli* PduQ-WT and ADH7-WT, respectively.

The recombinant *E*. *coli* strains were then grown in 100ml LB medium of 500 mL flask containing 40 μg/mL Ampicillin under aerobic conditions at 37°C with shaking at 220 rpm. The gene expression was induced by addition of 0.1mM IPTG when the bacterial growth had reached mid-exponential phase (OD_620_ around 0.5) with subsequent incubation for 24 h at 15°C and 160 rpm. The cells were then harvested and washed with 20 mM sodium phosphate buffer pH 7.4 containing 0.5 M NaCl.

Lysis of the cells was performed by re-suspending and incubating the cells in 5 ml Bugbuster protein extraction reagent and 10 μL lysonase bioprocessing reagent (Novagen) according to manufacturer´s instructions. The soluble protein fraction was separated from cell debris by centrifugation for 40 min at 16 000 x g, 4°C, and subjected to immobilized metal ion affinity chromatography (IMAC) for purification of the recombinant enzymes. The enzyme solution was loaded on 5 ml Ni-NTA HisTrap™ FF crude column equilibrated with binding buffer at a flow rate of 5 mL/min. After washing the column with the equilibration buffer, the bound protein was eluted using elution buffer containing 500 mM imidazole solution.

The eluted enzyme was concentrated 10 fold at 4°C in Vivaspin 15R (MWCO 10kDa) centrifugal concentrator tubes using a Sigma 3-16PK centrifuge at 3000 g for about 60 minutes. The concentrated enzyme solution (volume≈1mL) was mixed with 900 μL of pure glycerol and 20 μL of antioxidant stock solution (1M ascorbic acid, 50 mM copper sulphate), and stored in an airtight anaerobic glass vial at -20°C. Purity of the recombinant PduQ and ADH7 was analysed by sodium dodecyl sulfate-polyacrylamide gel electrophoresis (SDS-PAGE) on a gel containing 12% acrylamide. The protein bands were stained with Coomassie Brilliant Blue R-250.

### Analyses

Cell growth was monitored at 600 nm using a Ultrospec 1000 spectrophotometer (Pharmacia Biotech) and correlated with cell dry weight (CDW) as described previously [12]. For CDW determination, the culture broths (5 mL) were harvested and centrifuged (4000 x g) in a pre-weighed tube. After removing the supernatant, cell pellets were dried overnight in an oven at 110°C and then weighed again. The difference in weight is then refered to the cell dry weight in 5 mL cell culture.

Glucose, glycerol, lactate, ethanol, 1,3-PDO and 3HP concentrations were determined by HPLC (JASCO, Tokyo, Japan) equipped with refractive index detector. Separation of the compounds was performed on Aminex HPX-87H chromatographic column using sulfuric acid (50 mM) as mobile phase. The modified colorimetric method of Circle et al. [27] was used for measuring 3HPA concentration using acrolein as standard [28].

The activity of PduQ and ADH7 was determined as described by Reid et al. [20] with some modification. Aldehyde reduction was measured by following the decrease in absorbance of NADH at a wavelength of 340 nm (Δε_340_^NADH^ = 6.22mM^-1^cm^-1^) in a reaction mixture containing 0.2 mM NADH, 100 mM 3-HPA and appropriate amount of enzyme in 50 mM potassium phosphate buffer pH 7.0, if not mentioned otherwise. For determining alcohol oxidation, the reaction mixture contained 5 mM NAD^+^, 500 mM 1,3-PDO and the enzyme in 50 mM Tris-HCl pH 9.0. All spectrophotometric measurements were made using UV-1650 PC Spectrophotometer (Shimadzu, Kyoto, Japan) at 30°C.

3-HPA was produced by biotransformation of glycerol in aqueous solution using resting cells of *L*. *reuteri* as described by Sardari et al. [29]. One Unit (U) of the enzyme activity is defined as the amount of enzyme catalyzing the formation or consumption of 1 μmol NADH per minute under the standard assay conditions. Kinetic parameters were obtained by non-linear curve fitting to the Michaelis-Menten equation v = V_max_[S]/(K_m_+[S]) using GraphPad Prism6 software (http://www.graphpad.com).

## Results

### Central metabolic analysis of redox balancing in *L*. *reuteri*

**Fig 1:**
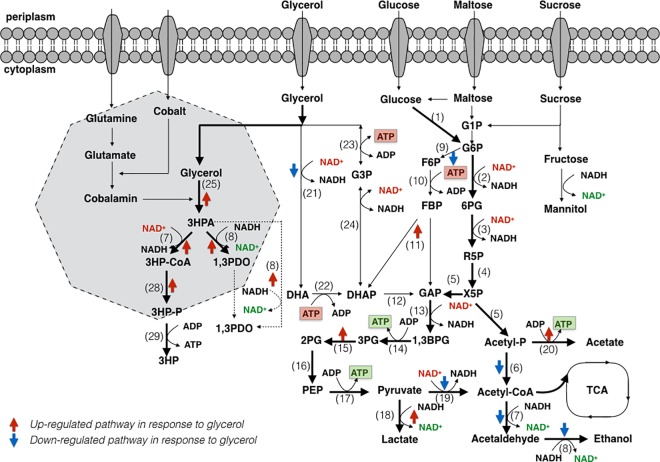
Pathways of glucose and glycerol metabolism in *L*. *reuteri* DSM20016. **A) The proposed routes for glucose metabolism using Embden- Meyerhof (EMP) and -phosphoketolase (PKP) pathways, and model of the Pdu microcompartment and -pathway for glycerol transformation. The enzymes involved are indicated as numbers, which are listed in Table 1.** Abbreviations: G1P, glucose-1-phosphate; 6PG, 6-phosphogluconate; X5P, xylulose-5-phosphate; Acetyl-P, acetyl phosphate; FBP, fructose-1,6-bisphosphate; GAP, glyceraldehyde-3-phosphate; 3PG, 3-phosphoglycerate; G6P, glucose-6-phosphate; 2PG, 2-phosphoglycerate; R5P, ribulose-5-phosphate; PEP, Phosphoenolpyruvate; G3P, glycerol-3-phosphate; 1,3BPG, 1,3-biphosphoglycerate; F6P, fructose-6-phosphate; DHAP, dihydroxyacetone phosphate; Adh, alcohol dehydrogenase; Aldh, aldehyde dehydrogenase; Ldh, lactate dehydrogenase; Gld, glycerol-2-dehydrogenase; G6pd, glucose-6-phosphate dehydrogenase; Gpd, glycerol-3-phosphate dehydrogenase; Gapdh, glyceraldehyde phosphate dehydrogenase; Pgdh, 6-phosphogluconate dehydrogenase; PDC, pyruvate dehydrogenase complex.

We reconstructed a stoichiometric metabolic pathway capturing the central metabolism of *L*. *reuteri* (Fig 1). *L*. *reuteri* is proposed to employ two glycolytic pathways to channel carbohydrates into the typical three-carbon intermediates [30]. In general, homofermentative LAB convert carbohydrates into lactate using the Embden-Meyerhof pathway (EMP), whereas heterofermentative LAB produce lactate, acetate and ethanol using the phosphoketolase pathway (PKP) by the enzymes listed in Table 1.

**Table 1:** A list of enzymes involved in the pathways of glucose and glycerol metabolism in *L*. *reuteri* DSM20016.

No.	Entry	Protein name	Locus	Length	Mass	exp^a^	sta	Sta-exp
1	A5VKT9	Hexokinase	lreu_1206	323	34421	-	-	-
2	A5VMD0	Glucose-6-phosphate dehydrogenase	lreu_1765	493	56374	-	-	-
3	A5VMD1	6-Phosphogluconate dehydrogenase	lreu_1766	478	53399	-	-	-
4	A5VKQ0	Ribulose-5-phosphate-3-epimerase	lreu_1167	217	23422	-	-	-
5	A5VM51	Phosphoketolase	lreu_1686	803	91404	-	-	-
6	A5VIJ3	Phosphate acetyltransferase	lreu_0398	324	34700	-1.22	-0.99	-1.18
7	A5VIB7	Aldehyde dehydrogenase	lreu_0321	878	97189	-2.01	-6.18	-7.18
7	A5VMA0	Aldehyde dehydrogenase (PduP)	lreu_1735	477	51127	0.81	-	0.82
8	A5VMM4	Alcohol dehydrogenase (ADH1)	lreu_1860	336	35918	-	-1,97	-
8	A5VM35	Alcohol dehydrogenase (ADH2)	lreu_1670	328	34921	-	-	-
8	A5VLU6	Alcohol dehydrogenase (ADH3)	lreu_1578	368	39107	-	-	-
8	A5VLQ6	Alcohol dehydrogenase (ADH4)	lreu_1535	348	37899	-	-	-
8	A5VLL8	Alcohol dehydrogenase (ADH5)	lreu_1496	342	36124	-0.87	-2.22	-2.95
8	A5VIB7	Alcohol dehydrogenase (ADH6)	lreu_0321	878	97189	-2.01	-6.18	-7.18
8	A5VHI2	Alcohol dehydrogenase (ADH7)	lreu_0030	390	42181	1.43	1.52	1.69
8	A5VLV6	Alcohol dehydrogenase (ADH8)	lreu_1589	351	37668	-	-	-
8	A5VM99	Alcohol dehydrogenase (PduQ)	lreu_1734	373	40361	0.90	0.62	0.90
9	A5VIL5	Phosphoglucose isomerase	lreu_0420	452	50351	-0.96	-	-
10	ND^b^	Phosphofructokinase	-	-	-	-	-	-
11	A5VI34	Fructose-bisphosphate aldolase	lreu_0238	288	31603	-	3.73	3.96
12	A5VHW7	Triose-phosphate isomerase	lreu_0171	256	28377	-	-	-
12	A5VI33	Triose-phosphate isomerase	lreu_0237	259	28515	-	-	-
13	ND	Glyceraldehyde phosphate dehydrogenase	-	-	-	-	-	-
14	A5VKB1	Phosphoglycerate kinase	lreu_1025	278	30347	-	-	-
15	A5VI87	Phosphoglycerate mutase	lreu_0291	236	27388	-	-	-
15	A5VI35	Phosphoglycerate mutase	lreu_0239	218	24664	-	3.69	3.67
15	A5VJ49	Phosphoglycerate mutase	lreu_0606	218	24793	-	1.21	-
15	A5VLZ3	Phosphoglycerate mutase	lreu_1627	217	24287	-	1.51	1.48
15	A5VM95	Phosphoglycerate mutase	lreu_1730	214	24822	-	1.56	2.20
16	ND	Phosphopyruvate hydratase (enolase)	-	-	-	-	-	-
17	A5VJJ1	Pyruvate kinase	lreu_0751	473	51826	-	-	-
18	A5VJZ6	Lactate dehydrogenase	lreu_0907	324	35005	-	-	-
18	A5VJF6	Lactate dehydrogenase	lreu_0716	319	34055	-	-	-
18	A5VMR6	Lactate dehydrogenase	lreu_1903	316	34793	-	-	-
18	A5VL05	Lactate dehydrogenase	lreu_1272	312	33353	-	-	-
18	A5VHZ0	Lactate dehydrogenase	lreu_0194	310	33972	0.94	-	1.85
18	A5VJF5	Lactate dehydrogenase	lreu_0715	358	38931	-	-	1.12
19	A5VJ72	Pyruvate dehydrogenase complex (E1-alpha)	lreu_0631	368	41003	-3.17	-	-
19	A5VJ73	Pyruvate dehydrogenase complex (E1-beta)	lreu_0632	325	35243	-3.26	-	-
19	A5VJ74	Pyruvate dehydrogenase complex (E2)	lreu_0633	444	48372	-3.36	-	-
19	A5VJ75	Pyruvate dehydrogenase complex (E3)	lreu_0634	475	50726	-3.28	-	-
20	A5VJ04	Acetate kinase	lreu_0560	398	43596	-	0.7	-
21	A5VMK5	Glycerol-2-dehydrogenase	lreu_1840	373	40699	-1.74	-3.42	-3.6
22	A5VKP6	Dihydroxyacetone kinase (Dak phosphatase)	lreu_1163	569	61416	-	-	-
23	A5VKE8	Glycerol kinase	lreu_1065	500	55389	-	-	-
24	A5VIG6	Glycerol-3-phosphate dehydrogenase	lreu_0371	338	36905	-	-	-
25	A5VMB2	Glycerol dehydratase I	lreu_1747	558	62092	0.71	0.88	1.18
25	A5VMB1	Glycerol dehydratase II	lreu_1746	236	25808	-	0.72	-
25	A5VMB0	Glycerol dehydratase III	lreu_1745	172	19319	0.8	0.73	1
27	A5VMA0	Propionaldehyde dehydrogenase	lreu_1735	477	51127	0.81	-	0.82
28	A5VMA5	Phosphate propanoyltransferase	lreu_1740	214	23962	0.73	-	-
29	A5VM98	Propionate kinase	lreu_1733	394	43803	-	-	-

^a^ Genes are differentially expressed in response to glycerol. Comparisons were established to identify mid-log (Exp), between mid-log and early-stationary growth phases (Sta-Exp), and early-stationary (Sta) specific responses. Differential as an absolute Log_2_(ratio(intensity of signal with glycerol/ intensity of signal without glycerol))>0.585 and a p-value<0.05 are defined (Data taken from [31]).

^b^ Genes that are not detected in genome of *L*. *reuteri*.

**Fig 2:**
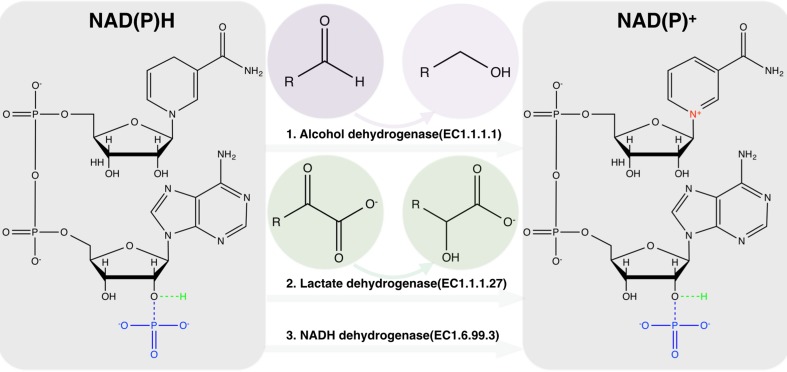
NAD(P)^+^ regeneration catalyzed mainly by three enzymes in *L*. *reuteri* (Alcohol dehydrogenase (EC1.1.1.1), Lactate dehydrogenase (EC1.1.1.27) and NADH dehydrogenase (EC1.6.99.3)). ADHs catalyse the NAD(P)^+^-dependent interconversion of aldehydes or ketones and enantiomeric alcoholic compounds. The complete structure of NAD^+^/NADH is indicated by a green H-atom, while the additional phosphate group of NADP^+^/NADPH is indicated in blue.

However, the relatively low growth rate as well as low biomass yield obtained in the presence of nonlimiting glucose concentration, indicate restriction in the growth of *L*. *reuteri*. Årsköld et al. [10] have earlier shown that the choice of carbon source could obviously affect the growth performance of *L*. *reuteri;* growth on 0.15 M sucrose resulted in a higher growth rate (0.82 h^-1^) and high ATP yield (10.5 g biomass•mol substrate^-1^), whereas growth with 0.28 M glucose was characterized by a maximum specific growth rate and ATP yield of about 0.45 and 5.2, respectively. In the present study, the growth limitation on glucose could be alleviated by the presence of glycerol, confirming that the limitation was imposed by a redox imbalance (Fig 1). With glycerol, part of the NAD(P)^+^ could be regenerated via the reduction of the 3-HPA to 1,3-PDO, using the activity of ADHs. As 3-HPA is transported outside the microcompartment into the cytoplasm, its reduction with concomitant regeneration of NAD^+^ could occur either via fermentation pathways by ADH and lactate dehydrogenase or instead by the passing of electrons to the electron transport chain and oxidation by NADH dehydrogenase (Fig 2).

### Alcohol dehydrogenases (ADHs) from lactobacilli

**Fig 3:**
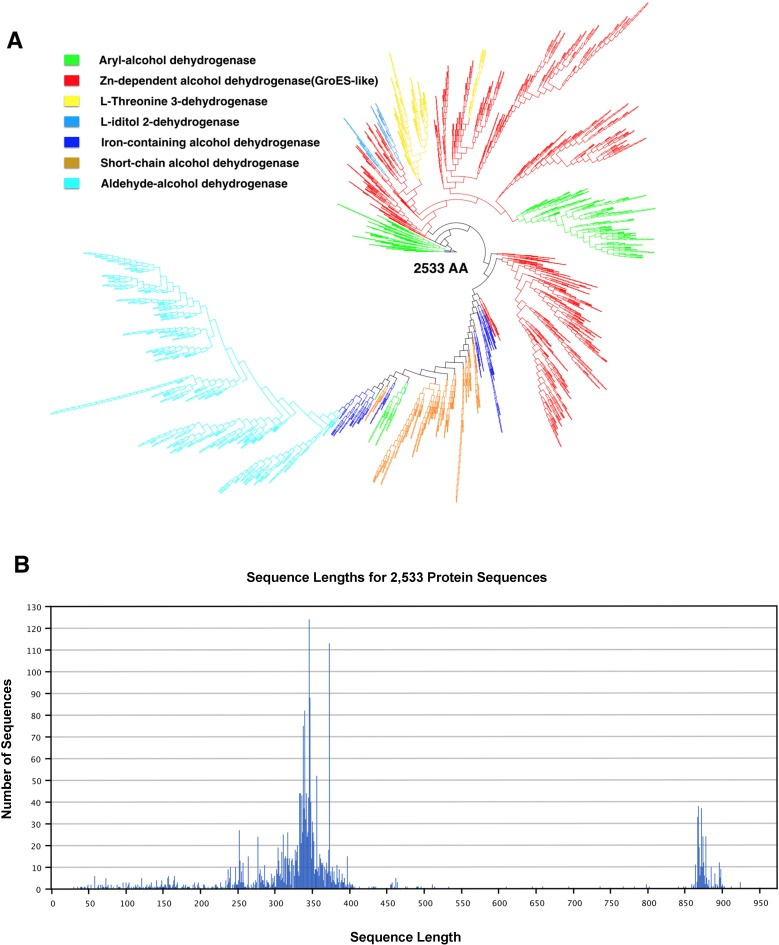
Phylogenetic tree derived from *Lactobacillus* species alcohol dehydrogenases and related protein amino acid sequences extracted from protein database Uniprot. The sequences (2533) were aligned using ClustalW and adjusted by gap insertion before being clustered using the NJ method with 100 bootstrap. Abbreviations are explained in S1 File.

To date, *Lactobacillus* is the largest genus of the LAB group, with over 50 species in total [32]. In order to analyse diversity amongst *Lactobacillus* ADHs and related proteins, all their amino acid sequences (2533 in number) available from public databases (UniProt, NCBI and PDB) were compiled and aligned using Geneious®9.1.2 for the purpose of constructing a phylogenetic tree using the average distance method and bootstrap analysis (Fig 3). The resulting phenogram revealed that within the NAD(P)-dependent group, the *Lactobacillus* ADHs are clustered in order according to enzyme type, i.e. zinc-dependent ADHs, short-chain ADHs, and iron-containning ADHs, irrespective of the species of *Lactobacillus*. It is worth mentioning at this point that ADHs from closely related species are often clustered together. Using this method, unknown *Lactobacillus* ADHs can be classified via phylogenetic relatedness into their respective ADH types, and as observed in the phylogenetic tree more than 70% of known *Lactobacillus* ADHs are grouped into Zn-dependent ADH type. Normally, zinc-containing long/medium-chain enzymes (group I ADHs) and metal-free short-chain enzymes (group II ADHs) are approximately 350 or 250 amino acids, respectively, in size, while the iron-containing ADHs (group III ADHs) exhibit a size of around 385 amino acid residues per subunit (Fig 3B). Typically, screening of all ADHs in *Lactobacillus* shows a group of ADHs in the range of 850 aa to 900 aa, referred as the bifunctional alcohol/aldehyde dehydrogenases.

All ADHs found in *L*. *reuteri* are listed in Table 1. A genome-wide transcription analysis of *L*. *reuteri* cells grown on glucose with differentially expressed genes in response to glycerol revealed 2.7 and 1.9 times higher expression of *ADH7* and *PduQ* genes in exponential phase, and during stationary phase the expression levels were 2.9 and 1.5 fold higher, respectively [31]. In contrast, *ADH5* and *ADH6* genes were down-regulated in the presence of glycerol. Particularly, *ADH6* gene expression dropped to 25% and 1% of the original in exponential and stationary phase, respectively, during glycerol feeding.

**Fig 4:**
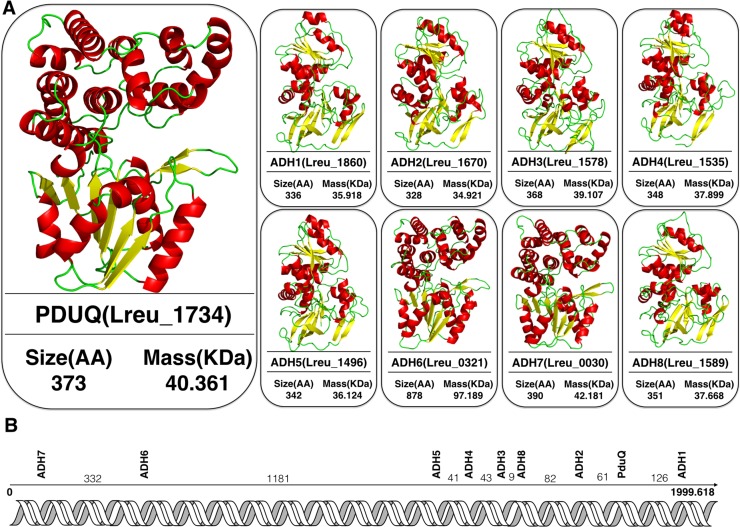
**A) Homology modeling of ADHs from *L*. *reuteri* DSM20016. B) Relative positions of the nine *L*. *reuteri* alcohol dehydrogenase (*ADH*) genes on the chromosome.** They are shown in the direction in which the genes are transcribed (arrows), the distances between the genes are indicted in kilobasepairs (kb).

All the *L*. *reuteri* ADHs are metal-ion dependent enzymes, where the metal-ion can have a catalytic and/ or a structure stabilizing effect on the enzyme. Addtionally, homology modeling for the prediction of the tertiary structure of these ADHs were obtained through the online sever Swiss-model and structural analysis were performed using Pymol (Fig 4). The modeled monomer of all ADHs contains about 320–400 amino acid residues except for the ADH6 with 878 aa residues. In general, the monomeric protein of ADH6 (alcohol dehydrogenase part), ADH7 and PduQ fold into two domains that are separated by a deep cleft. The N-terminal domain has a so-called Rossmann-fold architecture, which is a typical protein structure motif that binds nucleotide cofactor such as FAD^+^, NAD^+^ or NADP^+^. The C-terminal domain consists of *α-*helical structures, with an up-and-down bundle architecture well-known as a dehydroquinate synthase-like *α-*domain. Particularly, the binding site for cofactor NAD(P)^+^ resides in the deep hydrophilic pocket between the two domains. The parameters used for homology modeling and the outcome are listed in S2 Table. Our laboratory studies have shown these ADH enzymes to have low affinity and catalytic efficiency towards ethanol as substrate (Unpublished data). It has been speculated that most group III ADHs are mainly involved in aldehyde reduction in bacterial species rather than alcohol turnover. Nevertheless, the other alcohol dehydrogenases (ADH1-ADH5, and ADH8) in *L*. *reuteri* are more likely to play a role in ethanol transformation as reported in other microorganisms [33].

**Fig 5:**
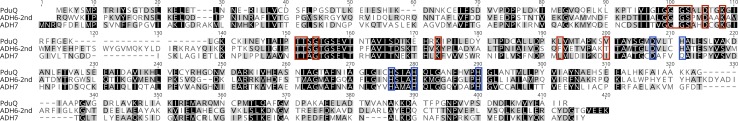
Sequence alignment of iron-dependent ADHs. The amino acid sequences are shown for truncated ADH6 (A5VIB7), ADH7 (A5VHI2) and PduQ (A5VM99) from *L*. *reuteri* DSM20016. The numbering scheme follows the amino acid sequence of ADHs. Identical residues in all sequences are highlighted in black and conserved residues in grey. The residues that interact with the NAD(P)^+^ cofactor and that coordinate with iron are enclosed by red and blue box, respectively.

To identify the genes involved in 3-HPA to 1,3-PDO conversion, a BLASTP analysis using PduQ as query was conducted among putative alcohol dehydrogenases in the genome of *L*. *reuteri* (NC_009513). The results indicated the presence of two other putative ADHs, i.e. ADH6 (lreu_0321, E-value: 2e^-53^) and ADH7 (lreu_0030, E-value: 5e^-46^). To identify features unique to these ADHs, protein sequence alignment of PduQ, ADH6 (truncated) and ADH7 was carried out after being aligned and adjusted by gap insertion. Similarities (Blosum62 with threshold 1) ranging from 42.9% to 48.4% were observed (Fig 5). The conserved amino acid sites marked by boxes revealed the common features for substrate/NADH/iron binding as in the iron-dependent ADHs. The specificity of all the enzymes towards NAD(P)^+^ is determined by motif G-G-G-S-X-X-D-X-X-K. In addition, five conserved metal-ion coordination residues, Asp207, His211, His276, His280 and His290 were found to be involved in the Fe^2+^ -binding.

### Characteristics of the ADH mutant *L*. *reuteri* cells

**Fig 6:**
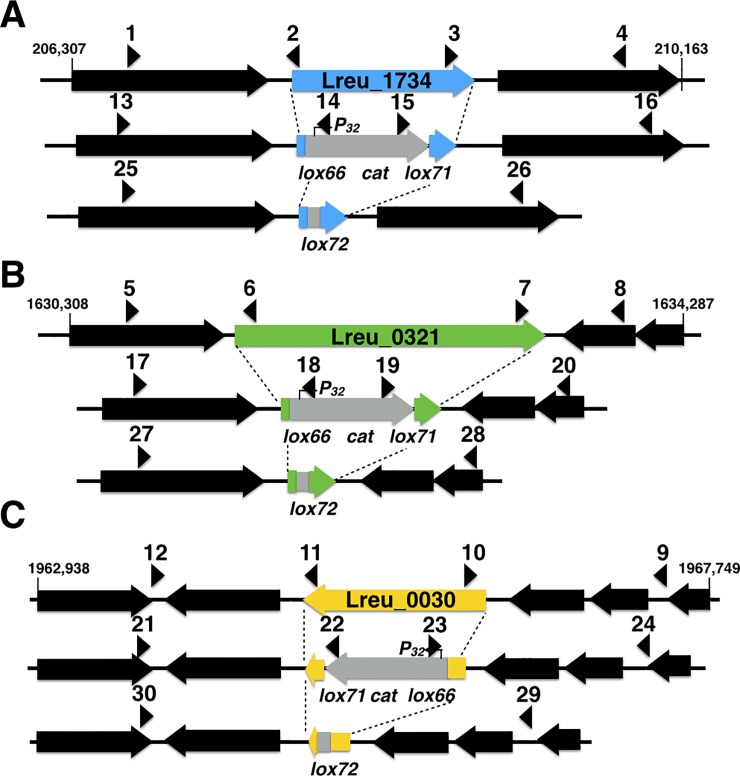
**Scheme for double-crossover mutant construction with removal of lreu_1734 gene (A), lreu_0321 gene (B) and lreu_0030 gene (C).** Firstly, the gene-specific mutagenesis vector was transformed into cells, after which the target gene was replaced by a *lox66-*P32-*cat-lox71* cassette according to homologous recombination. Then, the double-crossover mutant was selected by proper antibiotics, after which the *lox66-*P32-*cat-lox71* cassette would be resolved to a single double-mutant *lox72* site by transient *Cre* expression from the curable plasmid. Primers used were indicated as black arrowheads (see S1 Table).

To elucidate the iron-dependent alcohol dehydrogenase activity of ADH6, ADH7 and PduQ, three deletion strains were constucted using *Cre-lox*-based system resulting in strains with curing LCH010 (∆lreu_1734), LCH011 (∆lreu_0321) and LCH012 (∆lreu_0030), respectively (S1 Table). The strategy for construction of mutant for removal of lreu_1734 gene, lreu_0321 gene and lreu_0030 gene is illustrated in Fig 6.

**Fig 7:**
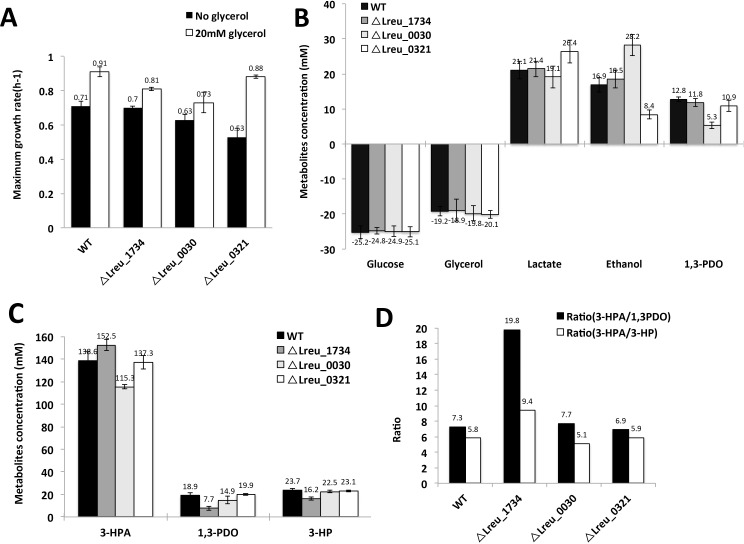
Growth and production characteristics of *L*. *reuteri* DSM20016 and its different mutants. (A) Maximum growth rate; (B) glycerol and glucose consumption and end-product formation in growing cells; (C) 3-HPA, 1,3-PDO and 3-HP production from 300 mM glycerol using resting cells; and (D) ratio of 3-HPA/1,3-PDO and 3-HPA/1,3-PDO during glycerol conversion by the resting cells. Mutant cells LCH010, LCH011, LCH012 involve deletions of PduQ, ADH6 and ADH7, respectively.

Addition of glycerol to mutant cells is expected to achieve regeneration of NAD(P)^+^ to various levels that in turn will have an impact on other coupled physiological reactions in the central metabolism. Therefore, tests on growth characterisitics of mutants were carried out by growing both wild type cells and mutants in MRS medium with or without glycerol supplementation. The growth rate without glycerol was observed to be similar for wild type (0.71 h^-1^) and LCH010 (0.70 h^-1^), but slightly decreased for LCH012 (0.63 h^-1^) and LCH011 (0.53 h^-1^) (Fig 7A). With the supplemented glycerol, the maximum growth rate of all cells increased by about 28%, 16%, 66% and 16% for wild type cells, LCH010, LCH012 and LCH011, respectively. Particularly for LCH012, the higher growth rate (0.88 h^-1^) suggests higher ATP production rate, and as a consequence end-product yield in this mutant can be partly replenished by the use of glycerol as the electron acceptor. Therefore, quantification of the metabolic end-products at an early stationary phase would provide more insight for the mutant behavior on NADH/NAD^+^ homeostasis.

As shown in Fig 7B, when grown in the presence of glycerol (20 mM), there was no notable difference in the glucose and glycerol comsumption by the wild type and all mutant strains. However, the clear differences were observed in end-product concentration, i.e. lactate, ethanol and 1,3-PDO. The wild type, LCH010 and LCH012 produced around 20 mM lactate, whereas LCH011 produced 26.4 mM lactate, which is 32% higher compared to the others. This could suggest that LCH011 had to resort to the use of LDH activity for regeneration of NAD^+^. More interestingly, nearly similar production of ethanol by wild type cells (16.9 mM) and LCH010 (18.5mM) was observed, while LCH012 produced relatively high amount of ethanol (28.2 mM) and ADH6 knockout strain LCH011 showed distinctly lower ethanol production (8.4 mM). This observation accords with the idea that ADH6 enzyme is a bifunctional alcohol/aldehyde dehydrogenase; similar enzymes are found in many other fermentative cells, and participate in ethanol generation by catalysing the conversion of an acyl-coenzyme A to an alcohol *via* aldehyde as an intermediate. Higher level of ethanol and lower 1,3-PDO production obtained in case of LCH012, obviously suggests the preference of the bifunctional ADH6 to regenerate NAD^+^ and also lower activity of PduQ for converting 3-HPA to 1,3-PDO. Indeed, up to 13 mM 1,3-PDO is produced by the wild type strain, LCH010 and LCH011, whereas production of 1,3-PDO in LCH012 was much lower (5.3 mM), which strengthened the assumption that ADH7 was most likely involved in the conversion of 3-HPA to 1,3PDO outside of Pdu microcompartment.

3-HPA, 3HP and 1,3-PDO production was then studied in a two-step process, in which *L*. *reuteri* cells were first cultivated anaerobically in MRS medium supplemented with 20 mM glycerol, followed by using the cells for transformation of 300 mM glycerol. The wild type and LCH011 strain produced nearly similar amounts of 3-HPA, i.e. 138.6 mM and 137.3 mM, respectively, while strain LCH010 produced 152.5 mM and LCH012 produced only 115.3 mM of 3-HPA (Fig 7C). The amounts of by-products 1,3-PDO and 3-HP formed by the wild type *L*. *reuteri* were 18.9 mM and 23.7 mM, respectively. The strain LCH010 produced 7.7 mM 1,3-PDO and 16.2 mM 3-HP, which results in a significantly higher ratio of 3-HPA/3-HP and 3-HPA/1,3-PDO as described in Fig 7D, showing that the ADH encoded by *PduQ* gene is mainly active in non-growing cells.

### Kinetic analysis of recombinant PduQ and ADH7 activities

Since PduQ and ADH7 seemed to catalyse 3-HPA reduction, both the enzymes were produced recombinantly and purified for further characterization. Studies with PduQ-His_6_ showed that the iron content of the enzyme (measured by ICP-MS) as well as the activity was lower in the enzyme exposed to air as compared to that under anaerobic conditions, confirming the iron dependence of the enzyne (unpublished data). Kinetic characterization of PduQ-His_6_ and ADH7-His_6_ with varied concentrations of aldehyde and alcohol substrates, respectively, obtained from GraphPad Prism6 showed the enzymes to exhibit significantly higher activity (K_cat_/K_m_) for aldehyde reduction than the oxidation of the corresponding alcohol, attributed to lower K_m_ as well as higher V_max_ with the aldehyde and NADH (Table 2). Propionaldehyde was a better substrate than 3HPA (not shown). Measurements at varying concentrations of cofactor with 3HPA or 1,3-propanediol as substrate revealed that the enzyme activity increased with increase in NADH or NAD^+^ concentration up to a certain extent. Maximum specific activity of PduQ-His_6_ for aldehyde reduction with NADH as cofactor (12.6± 0.7 U/mg) was about three-fold higher than that obtained for alcohol oxidation with NAD^+^ (3.9± 0.2 U/mg). On the other hand, the difference between the V_max-NADH_ and V_max-NAD+_ with the purified ADH7-His_6_ was eight-fold (33±2.1 vs 4.1±0.2 U/mg).

**Table 2:** Kinetic parameters for 3-HPA reduction/1,3-PDO oxidation by purified PduQ-His6 and ADH7-His6.

Enzymes	Reaction	Substrate	K_m_ (mM)	V_max_ (U/mg)	K_cat_ (s^-1^)	K_cat_/K_m_ (s^-1^.mM^-1^)
PduQ-His_6_	3-HPA reduction	NADH	0.04±0.01	12.6±0.7	8.5±0.5	237.44
		3-HPA	14.5±1.7	14.8±1.0	9.9±0.6	0.64
	1,3-PDOoxidation	NAD^+^	0.8±0.1	3.9±0.2	2.6±0.1	3.49
		1,3-PDO	(0.86±0.33)×10^3^	7.2±1.7	4.9±1.1	0.01
ADH7-His_6_	3-HPA reduction	NADH	0.04±0.12	33±2.1	11.3±1.9	282.5
		3-HPA	9.9±1.1	29.3±2.3	13.2±3.0	1.33
	1,3-PDOoxidation	NAD^+^	1.2±0.1	4.1±0.2	3.2±0.9	2.67
		1,3-PDO	233±8.5	10.4±1.1	2.8±1.0	0.01

## Discussion

Several microorganisms possess the ability to metabolize glycerol both reductively and oxidatively, e.g. *Klebsiella*, *Clostridium* or *Enterobacter* [34]. These species can utilize the NAD^+^-dependent enzyme glycerol dehydrogenase for the production of dihydroxyacetone (DHA), followed by subsequent phosphorylation to DHAP that gets funneled into glycolysis. However, *L*. *reuteri* does not grow on glycerol as the sole carbon and energy source, apparently due to an inactive glycerol dehydrogenase (lreu_1840, Fig 1). This assumption could also be confirmed by functional genomics analysis carried out by Santos *et al*. [31]. In their work, glycerol dehydrogenase gene was observed to be down-regulated 3.3-fold in the cells grown in the presence of glycerol compared to that without glycerol, and the extent of down-regulation was almost 10.8-fold once the cells entered early stationary (non-growth) phase.

*L*. *reuteri* possesses theoretically an alternative glycerol-utilization pathway involving initial reactions catalyzed by glycerol kinase (lreu_1065) and glycerol-3-phosphate dehydrogenase (lreu_0371), and the obtained dihydroxyacetone phosphate (DHAP) is converted to intermediate glyceraldehyde 3-phosphate by triose-phosphate isomerase (lreu_0171, lreu_0237) before entering the glycolytic pathway (Fig 1). However, no significant evidence has been found so far to prove the feasibility of this pathway in *L*. *reuteri*; a possible reason could be the reversible redox conversion catalyzed by the key enzyme glycerol-3-phosphate dehyderogenase (GPDH), which plays a vital role in lipid biosynthesis and has been shown to allow the prompt dephosphorylation of glycerol 3-phosphate into glycerol [35].

Accorrding to Talarico et al. [5], addition of glycerol (20 mM) reduced the generation time of *L*. *reuteri* from 66 min to 42 min, and as a consequence increased the final cell mass yield about 3-fold. Although further increase in glycerol concentration to 40 mM did not influence the generation time, the cell mass yield was consistently increased. Indeed, supplementation of the growth medium with glycerol leads to an obvious increase in acetate and a significant decrease in ethanol production, as also observed in our study.

These observations suggest that the Pdu pathway is the important route for glycerol metabolism in *L*. *reuteri*, and that glycerol, serving as an electron acceptor, could compete with acetyl phosphate in glucose fermentation, resulting in a shift in metabolite production from ethanol to acetate and the accumulation of 1,3-PDO (Fig 1).

Earlier studies have indicated that an alcohol dehydrogenase encoded by the Pdu operon is involved in glycerol/1,2-PD metabolism in the microcompartment in *L*. *reuteri* as well as in *Salmonella enterica*, and the role of the enzyme is mainly to regenerate NAD(P)^+^ from NAD(P)H [13,36]. It has also been shown that *L*. *reuteri* is able to excrete large amounts of 3-HPA, an antimicrobial compound, to the outside of microcompartment and into the extracellular medium while still maintaining viability at concentrations that are inhibitory to other microorganisms [37]. Hence, the self-defence mechanisms by 3-HPA detoxification in *L*. *reuteri*, as the producer strain, was assumed to exist by habouring other conversion routes of 3-HPA.

Some microorganisms have mutiple ADHs, sometimes of different types for different functions. For example, there is one well-characterised and 12 putative ADHs of one type found in *Sulfolobus solfataricus* [38], while *Pyrococcus furiosus* has two different types of ADHs [39]. The presence of mutiple ADHs within one organism conceivably reflects the environment to which the organism has been exposed and adapted. Therefore, each ADH may have a different role and specificity for survival.

In this study, we have identified nine different genes encoding alcohol dehydrogenases in *L*. *reuteri* DSM20016, three of which were hypothesized as genes encoding iron-dependent ADHs including PduQ present in the Pdu microcompartment. Deletion mutants of *L*. *reuteri* incorporating gene deletions of the iron-dependent ADHs ORFs (*PduQ*, *ADH6*, *ADH7*), respectively, displayed different behaviours in the production of metabolites. Supplementation with glycerol results in a higher growth of both *L*. *reuteri* wild type strain and mutants to different extents, but with little effect on glucose consumption. The mutants LCH010 (∆lreu_1734, PduQ deletion mutant) and LCH012 (∆lreu_0030, ADH7 deletion mutant) displayed 15.7% and 15.8% increased growth rate, respectively, in the presence of glycerol, compared to the wild type with 28% higher growth, implying an affected NAD^+^ regeneration for 3-HPA reduction (Fig 7). Most strikingly, LCH011 (∆lreu_0321, ADH6 mutant) displayed 66% increased growth rate with glycerol. Due to the lowest growth rate without glycerol (0.53 h^-1^) and almost similar growth as the wild type *L*. *reuteri* in the presence of glycerol (0.88 h^-1^), ADH6 is not likely to be involved in 3-HPA reduction but is responsible for another reaction in glucose fermentation.

Indeed, shift in metabolites production was further detected in the wild type *L*. *reuteri* and mutants. Although all the strains showed similar comsumption behaviour for glucose and glycerol, LCH012 produced 7.5 mM less 1,3-PDO but 11.3 mM higher ethanol concentration compared to the wild type (Fig 7). What was most suprising was that LCH010, the *PduQ* deletion mutant, displayed similar production behaviour as the wild type. In contrast, the role of ADH6, shown by LCH011 mutant, seems to be different. With 25.1% increased production of lactate and 50.5% reduced ethanol production, ADH6 is assumed to play a role in cofactor regeneration in the last step of glucose fermentation by converting acetyl-CoA to ethanol instead of 3-HPA conversion.

Previous genetic tests illustrated that a *PduQ* deletion mutant grew slower than wild-type *Salmonella* on 1,2-PDO and could not be complemented by co-expressing a non-microcompartment associated Adh2 from *Zymomonas* sp. that catalyzes the same reaction [36]. This phenotype proposes that PduQ keeps a microcompartment-specific function. The overarching function of Pdu microcompartment is to optimize metabolic pathways by restricting the efflux of toxic or volatile intermediates while allowing enzyme substrates, products and cofactors to pass [40]. In another report, Cheng et al. reported that it could be possible to restore the growth of a *PduQ* deletion mutant to wild-type levels by genetically disrupting the MCP shell [36], which was ascribed to facilitating the free diffusion of the metabolites and NAD^+^/NADH recycling.

The bifunctional alcohol/aldehyde dehydrogenase (ADHE) enzymes, which catalyse the conversion of an acyl-coenzyme A to an alcohol via an aldehyde intermediate, by coupling with oxidation of two NADH molecules to maintain the NAD^+^ pool during fermentative metabolism, are found in many microorganisms [41]. In *L*. *reuteri*, ADH6 is the bifuctional dehydrogenase, which seems to catalyse oxidation of acetyl-CoA to ethanol, and has little activity with 3-HPA substrate. The ADH-domain structure of ADH6 enzyme has been understood to consist of an Rossmann-fold architecture and an *α-*helical domain containing residues coordinating a metal ion, which is structurally similar to ADH7 and PduQ as shown by the homology-model. In accordance with the data for the *E*. *coli* ADHE [42], the activity of the *L*. *reuteri* ADH6 is only stimulated by the presence of Fe^2+^ and not by other metal ions (unpublished data), which is also the case for enzymes reported for *Streptococcus bovis* and *Entamoeba histolytica* [43,44].

The exact mechanism of iron-dependent alcohol dehydrogenases catalysed reduction of 3-HPA to 1,3-PDO with concomitant NAD^+^ generation remains to be elucidated. Nevertheless, the divalent metal ion in active site of the metal-dependent alcohol dehydrogenase (ADH) is believed to aid polarization of the aldehyde carbonyl O atom, facilitating reduction by NADH to proceed [37]. Furthermore, due to differences in the strength of the carbonyl group polarization, the charge density of the metal ion can have an effect on the catalysis rate [42].

Cumulatively, our studies have confirmed that the PduQ enzyme can convert NADH to NAD^+^ to supply the cofactor needed for oxidation of 3-HPA (in a reaction catalysed by PduP) internally within the MCP, while ADH7 is mainly responsible for providing NAD^+^ via 3-HPA to 1,3-PDO outside of the MCP. In fact, the latter enzyme exhibited faster reaction kinetics compared to PduQ (Table 2).

## Conclusions

The study reveals the diversity of alcohol dehydrogenases in *Lactobacillus* species, and their significance in energy and redox balance is exemplified in case of *L*. *reuteri* that uses glycerol as an electron acceptor and transforms it to 1,3-PDO via 3-HPA. Although glycerol is not used as a carbon source by *L*. *reuteri* for cell growth, it influences metabolism and improves growth through cofactor regulation; as the cofactor requirement is different for growing and resting cells, glycerol plays different roles. It is yet to be determined to what extent the distribution of energy carriers, such as NAD^+^/NADH through the microcompartment shells contribute to the regulation.

The findings presented here are the first to show the 3-HPA reduction to 1,3-PDO is not limited just to the activity of PduQ located in the bacterial microcompartment but also to ADH (ADH7) outside of the microcompartment, however with distinct roles in cofactor regeneration. Moreover, ADH6, the bifunctional alcohol/aldehyde dehydrogenase, does not play a role in 3-HPA reduction but is responsible for cofactor regeneration in the last step of glucose fermentation by converting acetyl-CoA to ethanol. Further studies on the individual enzymes are ongoing for gaining understanding of structure-function relationship as well as potential in biocatalysis.

## Supporting Information

S1 Fig Amino acid alignment of ADH1 (A5VMM4), ADH2 (A5VM35), ADH3 (A5VLU6), ADH4 (A5VLQ6), ADH5 (A5VLL8) and ADH8 (A5VLV6) from *L*. *reuteri* DSM20016. Red circle, putative catalytic residues; green circle, putative coenzyme binding motif; blue circle, the residues for the coordination of structural zinc. Strictly conserved residues are highlighted in black.
(TIF)

S2 Fig Schematic representation of mutagenesis vector pNZ5319. Indicated are the pACYC184-derived origin of replication (*ori*), the chloramphenicol resistance (Cm^r^) and erythromycin resistance (Em^r^) genes under control of P_32_ promoter, flanked by *lox66* and *lox71* site.
(TIF)

S3 Fig Homology modeling of the aldehyde domain of ADH6. (TIF)

S4 Fig a) Agarose gel analysis of DNA sequences of pET21a(B), *PduQ*(C) and pET21a:*PduQ*(D); b) SDS-PAGE analysis on 12% acrylamide gel of BL21(DE3):pET21a cell lysis(F), BL21(DE3):pET21a:*PduQ* cells lysis(G) and purified PduQ-His_6_(H); Standard nucleotide and protein ladder are shown in lane A and E, respectively; c) SDS-PAGE analysis on 12% acrylamide gel of BL21(DE3):pET21a cell lysis(2), BL21(DE3):pET21a:*ADH7* cells lysis(3) and purified ADH7-His_6_(4).
(TIF)

S1 File List of 2533 ADHs from *Lactobacillus* species. (XLS)

S2 File Phylogenetic tree derived from *Lactobacillus* ADHs with detailed information. (NEWICK)

S1 Table Stains, plasmids and primers used in this study. Cm^r^, chloramphenicol resistant; Em^r^, erythromycin resistant.
(DOCX)

S2 Table The document list of the results for homology modeling submitted to SWISS-MODEL workspace. (DOCX)
