# Supplementary figures and images for: Redox Balance in Lactobacillus reuteri DSM20016: Roles of Iron-Dependent Alcohol Dehydrogenases in Glucose/ Glycerol Metabolism
Source: PLoS One. 2016 Dec 28;11(12):e0168107. doi: 10.1371/journal.pone.0168107 (PMC5193401; doi:10.1371/journal.pone.0168107)

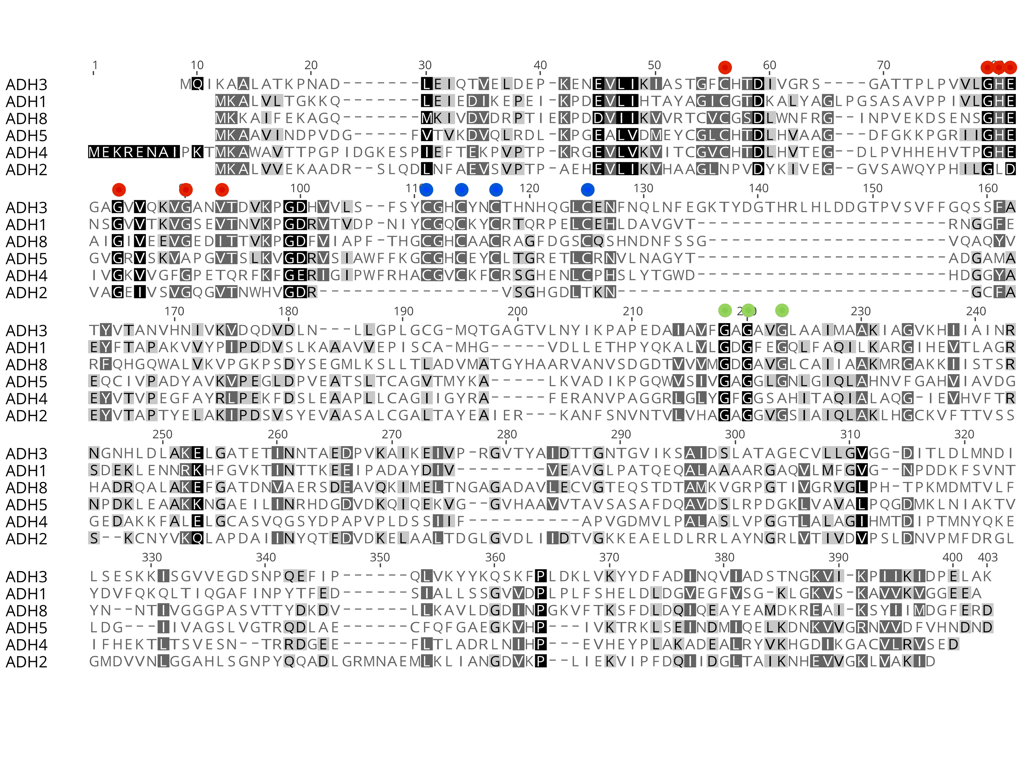

Supplement: S1 Fig — Red circle, putative catalytic residues; green circle, putative coenzyme binding motif; blue circle, the residues for the coordination of structural zinc. Strictly conserved residues are highlighted in black. (TIF) [file pone.0168107.s001.tif]

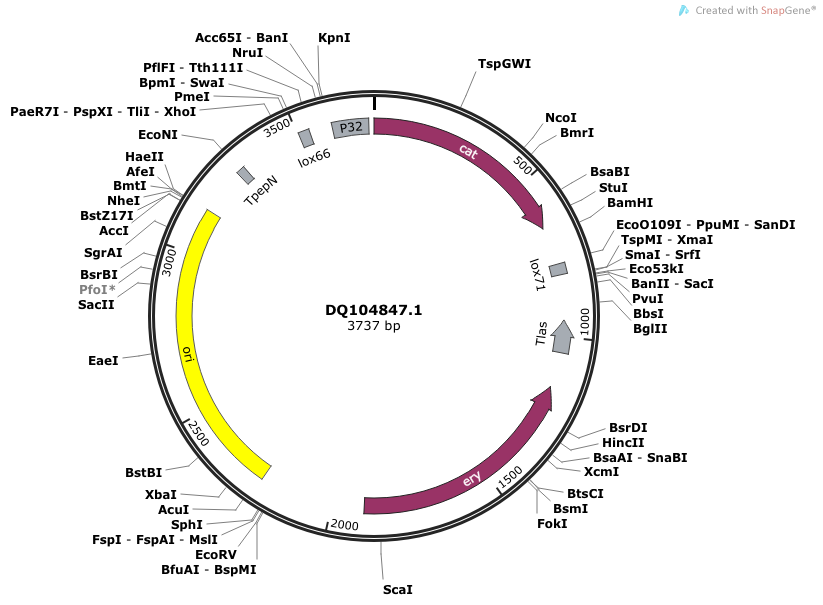

Supplement: S2 Fig — Indicated are the pACYC184-derived origin of replication (ori), the chloramphenicol resistance (Cmr) and erythromycin resistance (Emr) genes under control of P32 promoter, flanked by lox66 and lox71 site. (TIF) [file pone.0168107.s002.tif]

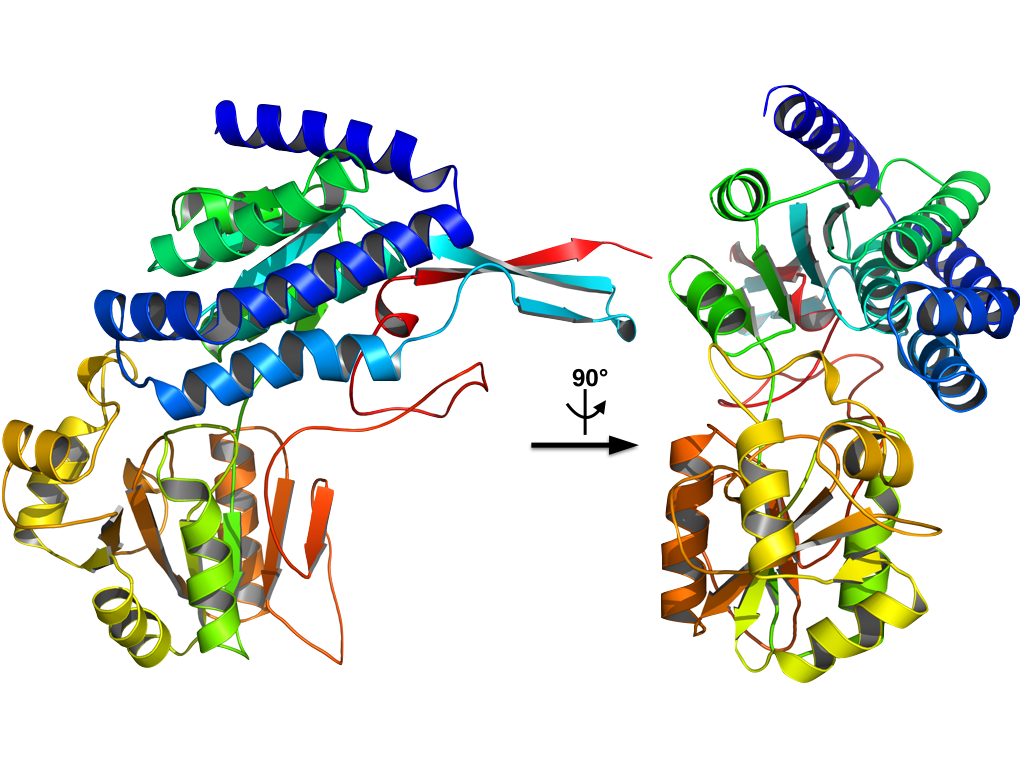

Supplement: S3 Fig — (TIF) [file pone.0168107.s003.tif]

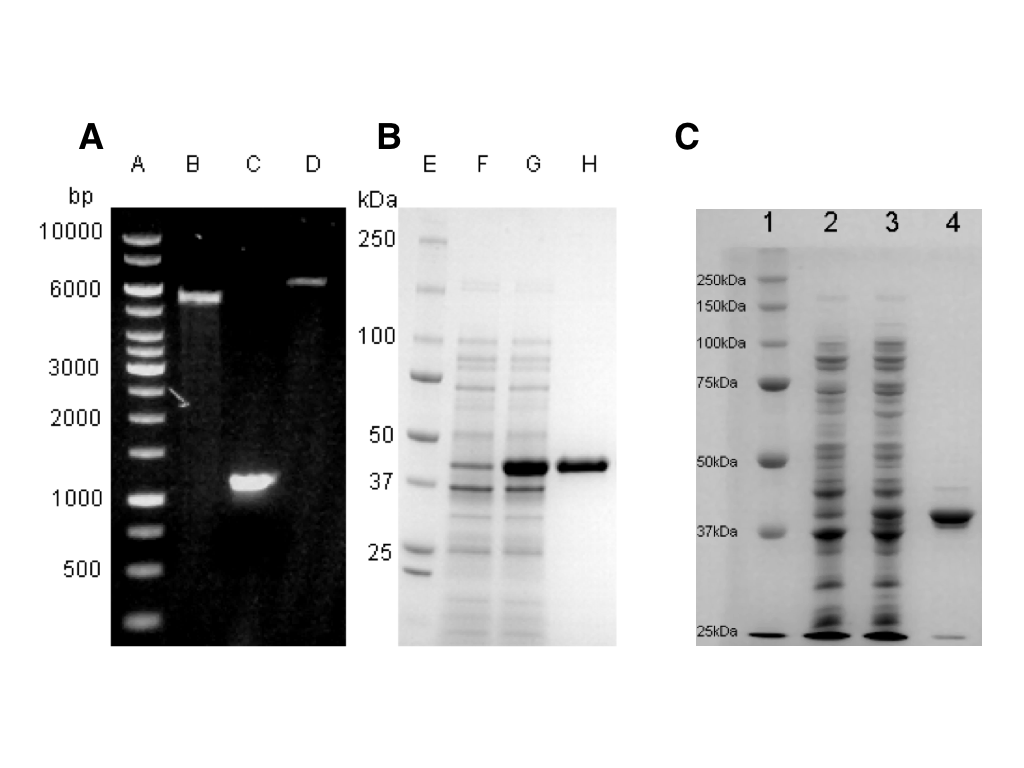

Supplement: S4 Fig — a) Agarose gel analysis of DNA sequences of pET21a(B), PduQ(C) and pET21a:PduQ(D); b) SDS-PAGE analysis on 12% acrylamide gel of BL21(DE3):pET21a cell lysis(F), BL21(DE3):pET21a:PduQ cells lysis(G) and purified PduQ-His6(H); Standard nucleotide and protein ladder are shown in lane A and E, respectively; c) SDS-PAGE analysis on 12% acrylamide gel of BL21(DE3):pET21a cell lysis(2), BL21(DE3):pET21a:ADH7 cells lysis(3) and purified ADH7-His6(4). (TIF) [file pone.0168107.s004.tif]
