# Supplementary material for: Redox Balance in Lactobacillus reuteri DSM20016: Roles of Iron-Dependent Alcohol Dehydrogenases in Glucose/ Glycerol Metabolism
Source: PLoS One. 2016 Dec 28;11(12):e0168107. doi: 10.1371/journal.pone.0168107 (PMC5193401; doi:10.1371/journal.pone.0168107)
Supplement: S1 Table — Cmr, chloramphenicol resistant; Emr, erythromycin resistant. (DOCX) [file pone.0168107.s007.docx]

|  | ***Material*** | ***Features*** | ***Source*** |
| --- | --- | --- | --- |
| **Strains** |  |  |  |
|  | *E.coli* |  |  |
|  | DH5a | Cloning host | lab collection |
|  | BL21(DE3) | Expression host |  |
|  | *L.reuteri* |  |  |
|  | DSM20016 | Wild-type strain | lab collection |
|  | LCH007 | Derivative of DSM20016 containing a *lox66*-P_32_-*cat-lox71* replacement of Lreu_1734 gene | this work |
|  | LCH008 | Derivative of DSM20016 containing a *lox66*-P_32_-*cat-lox71* replacement of Lreu_0321 gene | this work |
|  | LCH009 | Derivative of DSM20016 containing a *lox66*-P_32_-*cat-lox71* replacement of Lreu_0031 gene | this work |
|  | LCH010 | Derivative of LCH007 containing a *lox72* replacement of Lreu_1734 gene | this work |
|  | LCH011 | Derivative of LCH008 containing a *lox72* replacement of Lreu_0321 gene | this work |
|  | LCH012 | Derivative of LCH009 containing a *lox72* replacement of Lreu_0031 gene | this work |
| **Plasmids** |  |  |  |
|  | pET21a | Expression vector | Promega |
|  | pET21a:pduQ-His_6_ | pET21a vector with PduQ | This study |
|  | pET21a:ADH7-His_6_ | pET21a vector with ADH7 | This study |
|  | pNZ5319 | Cm^r^ Em^r^; pACYC184 derivative containing the *cat* gene under the control of P_32_ | lab collection |
|  | pNZ5348 | Em^r^; Cre expression vector | lab collection |
|  | pLCH007 | Cm^r^ Em^r^; pNZ5319 derivative containing homologous regions up- and downstream of Lreu_1734 gene | this work |
|  | pLCH008 | Cm^r^ Em^r^; pNZ5319 derivative containing homologous regions up- and downstream of Lreu_0321 gene | this work |
|  | pLCH009 | Cm^r^ Em^r^; pNZ5319 derivative containing homologous regions up- and downstream of Lreu_0031 gene | this work |
| **Primers** |  |  |  |
| 1 | prLCH-1734UF | TGCTAAGTTAAACAATGCCTTG | this work |
| 2 | prLCH-1734UR | TAAATCCGGGTTGGCATACT | this work |
| 3 | prLCH-1734DF | AAGAATTAGCTGATACTGTTGTTGCA | this work |
| 4 | prLCH-1734DR | ATGTTCACCAATTCCGGCTG | this work |
| 5 | prLCH-0321UF | TCTTGATACTGTTCAATCAGAAGCA | this work |
| 6 | prLCH-0321UR | TCTTGTTGTTAGCAGGCATAATTGA | this work |
| 7 | prLCH-0321DF | ACCCAGTTGAACCACTTGTT | this work |
| 8 | prLCH-0321DR | ATAGTTGCTTTGATCTTCGGAGT | this work |
| 9 | prLCH-0030UF | TTTAAAATAGGCATACGAGGTTACC | this work |
| 10 | prLCH-0030UR | AAGAAGTTCACACTTGGCAT | this work |
| 11 | prLCH-0030DF | TGTTGAAGATATTGTAAAGCTTTATCA | this work |
| 12 | prLCH-0030DR | TGATTTTGCGCGTTTCCTC | this work |
| 13 | prLCH-1734CUF | AAGAAACAGGAATGGGAAC | this work |
| 14 | prLCH-1734CUR | TCACAAAAATCCAAAGTAACC | this work |
| 15 | prLCH-1734CDF | AAGTAATCGTTAAACTGTTGCCG | this work |
| 16 | prLCH-1734CDR | GTCTAAATTCTTACAGATCTGACTTCT | this work |
| 17 | prLCH-0321CUF | GGACTGTCGGACATGTTTTTGG | this work |
| 18 | prLCH-0321CUR | TGGTACCTTACTTAACTTCGCGA | this work |
| 19 | prLCH-0321CDF | GGCACGTTAGGAATCATTACCG | this work |
| 20 | prLCH-0321CDR | GATTGTTGGAATAGTTGCTTTGATCT | this work |
| 21 | prLCH-0030CUF | AATGGCATAAGAATTAAAGGATTTGA | this work |
| 22 | prLCH-0030CUR | TAACTTCGCGACCATCGG | this work |
| 23 | prLCH-0030CDF | GGTTCGGAAGGCACGTTAG | this work |
| 24 | prLCH-0030CDR | TAAAATCGGTTAAAAAGCTGATTTTGC | this work |
| 25 | prLCH-1734CII-F | AAGAAACAGGAATGGGAAC | this work |
| 26 | prLCH-1734CII-R | GTCTAAATTCTTACAGATCTGACTTCT | this work |
| 27 | prLCH-0321CII-F | GGACTGTCGGACATGTTTTTGG | this work |
| 28 | prLCH-0321CII-R | GATTGTTGGAATAGTTGCTTTGATCT | this work |
| 29 | prLCH-0030CII-F | AATGGCATAAGAATTAAAGGATTTGA | this work |
| 30 | prLCH-0030CII-R | TAAAATCGGTTAAAAAGCTGATTTTGC | this work |
|  | PduQF | ATTAGGATCCATGGAAAAATATAGTATGCCAACC | this work |
|  | PduQR | ATTACTCGAGACGAATTATTGCTTCGTAAACC | this work |
|  | ADH7F | ATGAATAGACAATTTGATTTCTTAA | this work |
|  | ADH7R | GTAGATGCCATCGTAAGC | this work |
