# Supplementary material for: Redox Balance in Lactobacillus reuteri DSM20016: Roles of Iron-Dependent Alcohol Dehydrogenases in Glucose/ Glycerol Metabolism
Source: PLoS One. 2016 Dec 28;11(12):e0168107. doi: 10.1371/journal.pone.0168107 (PMC5193401; doi:10.1371/journal.pone.0168107)
Supplement: S2 Table — (DOCX) [file pone.0168107.s008.docx]

| **Code Name** | **Template** | **Ligand** | **GMQE** | **Oligo-state** | **Seq**  **Identity** | **Seq**  **Similarity** | **Description** |
| --- | --- | --- | --- | --- | --- | --- | --- |
| ADH1 | 4ejm.1.A | zinc ion | 0.68 | homo-tetramer | 32.02 | 0.37 | zinc-binding dehydrogenase |
| ADH2 | 4dup.1.A | zinc ion | 0.65 | homo-dimer | 28.84 | 0.35 | quinone oxidoreductase |
| ADH3 | 1f8f.1.A | zinc ion | 0.75 | homo-tetramer | 46.01 | 0.43 | benzyl alcohol dehydrogenase |
| ADH4 | 1llu.1.A | zinc ion | 0.67 | homo-tetramer | 35.65 | 0.37 | alcohol dehydrogenase |
| ADH5 | 4eex.1.B | zinc ion | 0.82 | homo-dimer | 63,10 | 0.49 | alcohol dehydrogenase |
| ADH6-II | 3zdr.1.A | Fe ion | 0.79 | homo-dimer | 56.05 | 0.46 | alcohol dehydrogenase domain of the bifunctional acetaldehyde dehydrogenase |
| ADH7 | 4fr2.1.A | Fe ion | 0.88 | homo-dimer | 77.14 | 0.53 | 1,3-propanediol dehydrogenase |
| ADH8 | 4cpd.1.A | zinc ion | 0.67 | homo-dimer | 31.95 | 0.36 | alcohol dehydrogenase |
| PDUQ | 3zdr.1.A | Fe ion | 0.74 | homo-dimer | 39.89 | 0.39 | alcohol dehydrogenase domain of the bifunctional acetaldehyde dehydrogenase |
